# PP-Based Blends with PVP-I Additive: Mechanical, Thermal, and Barrier Properties for Packaging of Iodophor Pharmaceutical Formulations

**DOI:** 10.3390/polym17182442

**Published:** 2025-09-09

**Authors:** Melania Leanza, Domenico Carmelo Carbone, Giovanna Poggi, Marco Rapisarda, Marilena Baiamonte, Emanuela Teresa Agata Spina, David Chelazzi, Piero Baglioni, Francesco Paolo La Mantia, Paola Rizzarelli

**Affiliations:** 1 Institute of Polymers, Composites and Biomaterials-National Research Council (IPCB-CNR), 95126 Catania, Italy; melanialeanza@cnr.it (M.L.); domenicocarmelo.carbone@cnr.it (D.C.C.); marco.rapisarda@cnr.it (M.R.); emanuelateresaagata.spina@cnr.it (E.T.A.S.); 2 Department of Chemistry “Ugo Schiff” and CSGI, University of Florence, Sesto Fiorentino, 50019 Florence, Italy; poggi@csgi.unifi.it (G.P.); david.chelazzi@unifi.it (D.C.); piero.baglioni@unifi.it (P.B.); 3 Department of Engineering, University of Palermo, 90128 Palermo, Italy; marilena.baiamonte@unipa.it; 4 National Interuniversity Consortium of Materials Science and Technology (INSTM), 50121 Florence, Italy

**Keywords:** povidone-iodine, polypropylene, blends, iodophor, plastic packaging

## Abstract

The influence of minor components on leaching molecular iodine (I_2_) through polypropylene (PP)-based packaging from a povidone iodine-based (PVP-I) formulation, simulating an ophthalmic application, was evaluated. I_2_ is a cheap, broad-spectrum, and multi-target antiseptic. Nevertheless, it is volatile, and the prolonged storage of I_2_-based formulations is demanding in plastic packaging because of transmission through the material. Therefore, we explored the possibility of moderating the loss of I_2_ from an iodophor formulation by introducing small amounts of molecular iodine into the polymer material commonly used in eyedropper caps, i.e., PP. Thus, PP was blended via an extrusion process with a polymeric complex containing iodine (such as PVP-I) or with a second polymeric component able to complex the I_2_ released from an iodophor solution. The aim of this work was to introduce I_2_ into PP-based polymer matrices without using organic solvents and indirectly, i.e., through the addition of components that could generate molecular iodine or complex it in the solid phase, as I_2_ is heat-sensitive. To increase the miscibility between PP and PVP-I, poly(N-vinylpyrrolidone) (PVP) or a vinyl pyrrolidone vinyl acetate copolymer 55/45 (Sokalan) were added as compatibilizers. The PP-based binary and ternary blends, in granular or sheet form, were characterized thermally (Differential Scanning Calorimetry, DSC, and Thermogravimetric analysis, TGA), mechanically (tensile tests), morphologically (scanning electron microscopy (SEM)), and chemically (attenuated total reflectance Fourier transform infrared (ATR-FTIR)). Additionally, the variation in wettability induced by the introduction of the hydrophilic minority components was determined by static contact angle measurements (static contact angle (SCA)), and tests were carried out to determine the barrier properties against oxygen (oxygen transmission rate (OTR)) and molecular iodine. The I_2_ leaching of the different blends was compared with that of PP by monitoring the I_2_ retention in a buffered PVP-I solution via UV-vis spectroscopy. Overall, the experimental data showed the capability of the minority components in the blends to increase thermal stability as well as act as a barrier to oxygen. Additionally, the PP blend with PVP-I induced a reduction in molecular iodine leaching in comparison with PP.

## 1. Introduction

Molecular iodine (I_2_) is known to have strong bactericidal, fungicidal, and virucidal activities but low solubility in water. The volatility and toxicity at high concentrations have long limited its biomedical applications. The discovery of the ability of I_2_ to form complexes with natural and synthetic hydrophilic polymers, cyclodextrins, and surfactants has allowed overcoming these critical issues and extending its use in the medical and pharmacological fields [1,2,3]. The best known and currently used commercial “iodophor” is povidone-iodine (PVP-I), largely utilized as antiseptic, in medical and clinical settings for its broad-spectrum antimicrobial properties.

In the povidone-iodine complex, I_2_ is bound to poly(N-vinylpyrrolidone) (PVP) via hydrogen bonds between two pyrroles; the complex contains triiodide anions and a small amount of mobile, noncomplexed molecular iodine (I_2_-free), which is the active bactericidal agent [4,5]. The complex, when in an aqueous medium, releases free molecular iodine (I_2_) into the solution, which performs its bactericidal function [6]. It has been found that the amount of free I_2_ released and, consequently, the bactericidal activity are dependent on the concentration of PVP-I and, paradoxically, increase with increasing dilution of PVP-I solutions, which is required in ophthalmic applications [7].

Packaging, including the container and closure systems, is essential in preserving safety, efficacy, quality, and shelf life of ophthalmic products. All the packaging components act as protective barriers against environmental factors such as light, temperature, moisture, and contamination, which can significantly influence the stability and usefulness of ophthalmic formulations. The key requirements of packaging, containers, and closures can be necessary for ophthalmic products. The sensitivity of some formulations requires specific packaging design to maintain concentration stability and sterility, guarantee exact dosing, or enhance patient safety and compliance. Common materials for ophthalmic packaging include glass, polyethylene (PE), polypropylene (PP), polyethylene terephthalate (PET), polymers, aluminum, and multilayer packaging [8]. PVP-I solutions, as well as other molecular iodine formulations for medical use, have been packaged, e.g., in soft plastic bottles that can be used for various medical purposes. However, a serious problem that has arisen with these I_2_ solutions packaged in plastic containers is that molecular iodine has leaked from the packaging, compromising the efficacy and stability of the pharmaceutical product [9,10,11]. This especially has been observed for dilute PVP-I solutions used to treat sensitive organs such as the eyes [12,13]. In fact, the loss of I_2_ is a function of the packaging material for a given temperature and PVP-I concentration. The main function of packaging materials is to prevent product damage, such as from environmental hazards (light, heat, oxygen, moisture, microorganisms, atmospheric particles, and gases). Depending on the type of product, packaging materials must provide protection against the loss or uptake of water or oxygen as well as crossing of any volatile component, aroma scalping, and leaching of contents. Thus, packaging materials must be able to preserve the product throughout its shelf life [14].

Glass (not permeable to I_2_) is the most suitable material for the storage of dilute PVP-I solutions required in ophthalmic formulation. However, the stability of dilute solutions can be affected by the presence of plastic components (e.g., droppers) in the glass containers, which may be an important source of I_2_ leaching. Recently, the capability of calixarene derivative, embedded with polypropylene (PP) through a swelling procedure, to reduce I_2_ losses from an iodophor formulation has been demonstrated [15].

Polymer blending is a well-known and versatile strategy for developing a polymer material with outstanding performance by balancing or removing the fault of a single-component polymer. For packaging materials, high gas barrier polymers are used commonly as the dispersed phase to improve the barrier properties of the matrix. Overall, the gas barrier enhancement of the polymer blends depends on their composition, the morphology of the dispersed phase, as well as interfacial adhesion and interaction between the component phases. Interfacial compatibility between the different component phases in polymer blends is a key factor in determining their final performance, particularly for the gas barrier properties. Compatibilization is widely utilized simply by adding a compatibilizer to a polymer blend, increasing the interfacial adhesion, stability of morphology, and degree of dispersion, which are valuable for enhanced gas barrier performance [16]. In this study, we investigated the possibility of reducing I_2_ leaching through the packaging and improving oxygen permeability to preserve the integrity of the product by introducing small amounts of molecular iodine in the polymeric material commonly used in bottle droppers, i.e., polypropylene (PP). This was obtained via mechanical mixing with a polymeric complex containing molecular iodine, without the use of organic solvents (indirect introduction). To this end, PP was mixed with a polymeric complex containing I_2_ (such as PVP-I) or with a second polymeric component able to complex the I_2_ released from an iodophor solution (such as PVP or Sokalan). Sokalan, a vinyl pyrrolidone vinyl acetate 55/45 copolymer, was selected for its dual role in the blends. On the one hand, its PVP-like segment allows it to form a complex with I_2_, while on the other hand, its hydrophilic vinyl acetate component contributes to improving compatibility between different polymers. Overall, the aim of this work was to introduce molecular iodine into PP-based polymer matrices indirectly through the addition of components that could be able to generate I_2_ or complex it in the solid phase, since I_2_ is heat-sensitive and without organic solvents. PP was selected since it is the polymer material commonly used in eyedropper caps. To improve the miscibility between PP and PVP-I, compatibilizers, such as PVP and a vinyl pyrrolidone vinyl acetate copolymer, were added to the blends via an extrusion process. The PP-based blends were characterized by DSC and TGA, tensile tests, SEM, and ATR-FTIR. Moreover, the change in wettability induced by the introduction of the hydrophilic minority components was determined by means of static contact angle (SCA) measurements, and tests were carried out to determine the barrier properties against oxygen (OTR) and I_2_ leaching. All blends without Sokalan showed improved barrier properties for oxygen. The PP-based blend with PVP-I was found to be able to reduce the I_2_ leaching from dilute aqueous solutions of PVP-I. This new PP-based system is exclusively devoted to the preparation of packaging of iodophor pharmaceutical formulations for ophthalmic applications. The improved performance can be considered only for similar PP-based systems, with comparable applications and in the same experimental conditions (pH, temperature, stabilizers, etc.). In comparison with our previous results [15], the PP blended with PVP-I showed improved thermal stability and greater oxygen barrier properties than PP, with the valuable advantage of having been prepared without the use of solvent via a simple extrusion process.

## 2. Materials and Methods

### 2.1. Materials

**Figure 1:**
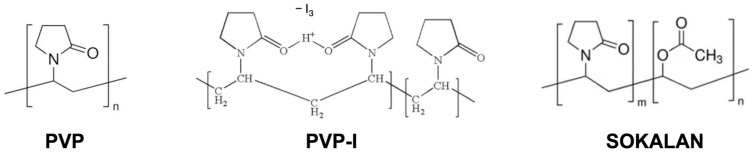
Structures of the repetitive units of the polymers used in the PP blends.

The starting polymers used for the preparation of the blends were PP (Purell HP371P, LyondellBasell, Milan, Italy), PVP K30 (Sigma-Aldrich Chemical Co., Ltd. Milan, Italy), PVP-I (PVP-Iodine 30/06 BASF, Milan, Italy), and Sokalan (VA 64 P 25 K BASF Milan, Italy), a vinyl pyrrolidone vinyl acetate copolymer 55/45 (Figure 1).

### 2.2. Sample Preparation

Polymer blends were prepared using a Dr. Collin Teach Line ZK 25T co-rotating twin-screw extruder with a screw diameter of 25 mm. The screws were equipped with specifically designed profiles to optimize the mixing of the polymers under investigation. Precise temperature control during extrusion was achieved through four independent heating zones. The temperature was set to 80 °C in the first zone, increased to 175 °C in the second one, and maintained at 180 °C in zones three and four, as well as at the extrusion die. The pressure at the extrusion die was held at 35 bar, the screw speed was held at 25 rpm, and the feeder rate was held at 25%. Cooling of the extruded filament was accomplished using forced air. Subsequently, the filament was fed into a pelletizer, where it was processed into granules of the desired composition, becoming ready for subsequent characterization. The composition and abbreviation of each polymer blend is shown in Table 1. Since our goal was to limit variations in the mechanical properties of PP, we chose to prepare blends that did not contain more than 4% minority components.

Samples for mechanical and permeability tests were prepared via compression molding using a Carver laboratory hydraulic press at 180 °C for 5 min. The thickness for all samples with PP matrix varied between 230 and 290 µm. The samples containing PVP-I showed an amber color, while the PP + 2% Sokalan and PP + 2% PVP samples exhibited a yellow coloration.

### 2.3. Characterization Techniques

#### 2.3.1. Scanning Electron Microscopy (SEM)

The analyses were carried out with a Phenom ProX desktop SEM (Thermo Fisher Scientific, Waltham, MA, USA), using an acceleration potential of 15 KV. For the fractured cross section analysis, the samples were cracked previously in liquid nitrogen. Analysis of the SEM images was performed using ImageJ software (v. 4.6.0).

#### 2.3.2. Attenuated Total Reflectance Fourier Transform Infrared (ATR-FTIR) Spectroscopy

The samples were analyzed using a Cary 620–670 FTIR microscope equipped with an MCT detector (Agilent Technologies, Santa Clara, CA, USA) and an ATR element (Germanium). For each measurement, 128 scans were acquired, with a spectral resolution of 4 cm^−1^ and a spectral range of 4000–400 cm^−1^. The spectra were analyzed using Agilent ResPro software (v. 5.4.1) and the IGOR64TM program (v. 9.0).

#### 2.3.3. Thermogravimetric Analysis (TGA)

Samples were analyzed using an SDT 650, which is capable of simultaneously acquiring TGA and DSC measurements (Ta Instruments/Waters, New Castle, DE, USA). For each measurement, approximately 8–12 mg of each sample was weighed. The sample was placed in open alumina pans and subjected to a thermal scan from room temperature to 600 °C, with a heating rate of 10 °C/min. Measurements were performed under a nitrogen flow set at 100 mL/min. Data were analyzed with TA Trios software (v. 5.1.1).

#### 2.3.4. Differential Scanning Calorimetry (DSC)

The samples, in the form of plates, were analyzed by a DSC 2500 (Ta Instruments/Waters). For each measurement, approximately 8–12 mg of each sample was weighed. The sample was placed in hermetically sealed Tzero aluminum pans (TA Instruments/Waters). The samples were cut into small portions of 4–6 mm^2^ and inserted into crucibles. We took care to arrange them to almost completely cover the surface of the container and ensure that all the pieces were stacked. The following temperature program was applied for each sample: a rapid descent to −90 °C, 10-min isotherm, first heating ramp at 10 °C/min up to 250 °C (to erase the thermal history of the samples), a second rapid descent to −90 °C, a second 10-min isotherm, and a second heating ramp at 10 °C/min up to 250 °C. The measurements were carried out under a nitrogen flow set to 50 mL/min. The experimental data were analyzed using TA Trios software. Data relating to the melting temperature (***Tm***) and the enthalpy of fusion (***ΔHm***), obtained by integrating the melting peak, as well as the degree of crystallinity (***Xc***) of the sample were obtained. The latter value was calculated using the ***ΔHm*** measured on the sample and that of a reference system with 100% crystallinity, which was obtained from the literature, according to Equation (1):

(1) $$Xc=\;\left(\frac{\Delta Hm}{\Delta H{}^{\circ}m}\right)\times \;100$$

For the PP and its blends, the value most reported in the literature for polypropylene was used, i.e., 207 J/g [17], which is currently under review [18].

#### 2.3.5. Mechanical Characterization

Stress–strain curves were measured using a universal testing machine mod. 3365 (Instron, Norwood, MA, USA). The elastic modulus was measured at a speed of 1 mm/min until the deformation was 10%. Then, the crosshead speed was increased to 100 mm/min until the specimen broke. The values of the elastic modulus ***E***, tensile strength ***TS***, and elongation at break ***EB*** were calculated as an average of 10 experimental values.

#### 2.3.6. Static Contact Angle (SCA) Measurements

A contact angle goniometer (OCA15EC, Dataphysics, Filderstadt, Germany) was used to measure the surface wettability values of the samples. Before measurement, the samples were equilibrated in an oven at 40 °C for 30 min to eliminate any interferences. The SCA values were determined at room temperature by dropping 2 μL of water onto the surfaces of the films using a microsyringe. The images taken by the connect camera were analyzed by using the SCA 20 software. The films were kept flat using a film sample holder, which allowed them to be positioned and stretched correctly. To ensure the reproducibility of the experiment, five measurements were carried out on each sample.

#### 2.3.7. Oxygen Permeability

The samples were tested at room temperature (25 °C) with a Systech Permeability Meter Illinois 8001 to measure the oxygen transmission rate (OTR), i.e., the amount of gas (O_2_) that passes through a unit surface of a defined thickness and under a given partial pressure difference per unit of time (with the unit of measurement being cm^3^ m^–2^ day^−1^). The test consisted of fixing the sample in the diffusion chamber and then introducing pure oxygen (99.9% gas test) into the upper part of the chamber while a carrier gas (nitrogen) without oxygen was flushed into the lower part. Oxygen molecules diffusing through the sample in the lower part of the chamber were carried to the sensitive sensor in the gas test, which recorded the increase in concentration over time. At the end of the delay time, the concentration from the gas test for the carrier gas in the lower half of the cell stabilized at a value proportional to the permeability of the material.

#### 2.3.8. UV-Vis Spectroscopy

UV-Vis spectra were recorded at room temperature with an Agilent Technologies 8453 UV-Vis spectrophotometer using 1-cm path length quartz cells and a Jasco v770 spectrophotometer equipped with an integrating sphere for reflectance measurements.

#### 2.3.9. Assessment of Molecular Iodine Leaching

**Figure 2:**
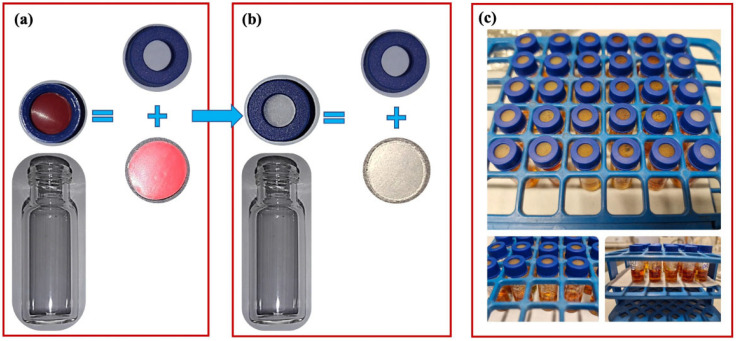
(**a**) Original and (**b**) modified GC vials and caps with the discs of PP and its blends. (**c**) Photographic documentation of PVP-I solutions in contact with air.

A comparative test was conducted to evaluate the barrier’s effect against I_2_ leaching through an ophthalmic formulation container and to assess the influence of minority components on I_2_ leaching compared to neat PP. For this purpose, discs were prepared from film samples (500 µm thick) made in a laboratory using a hot-pressing machine (PM 20/200 press—DGTS srl, Verduggio, Monza Brianza, Italy). Specifically, 5.5 g of pellets obtained from the different polymer blends was placed between two heating plates heated to a temperature of 180 °C for 5 min without pressure and then immediately pressed for 2 min at about 25 bar. After this time, the samples were cooled to 80 °C. The discs were cut from the film using a suitable die cutter. These discs, pre-selected for optimal sealing, were inserted into a gas chromatography vial cap, replacing the original septum to simulate the plastic dropper of a container (Figure 2a,b). Then, 0.8 mL of a 0.3% sodium citrate-buffered PVP-I solution (pH = 6) was added to the vial (Figure 2c) [15]. The accelerated test was carried out at 40 °C for 5 days. The amount of I_2_ present in the PVP-I solution was checked via extraction with 0.8 mL of cyclohexane at time zero and after 5 days and determined with UV-Vis spectroscopy at λ = 523 nm using a calibration curve. In addition, the changes in the UV-Vis reflectance spectra of the discs were monitored at time zero and after 5 days [15].

## 3. Results

The chemical, thermal, and morphological characterization, by means of ATR-FTIR, DTG, DSC, and SEM measurements, was carried out on portions of sheets prepared by compression molding and on the minor pure components—PVP, PVP-I, and Sokalan—using the same operating methods.

### 3.1. Structural and Morphological Properties

**Figure 3:**
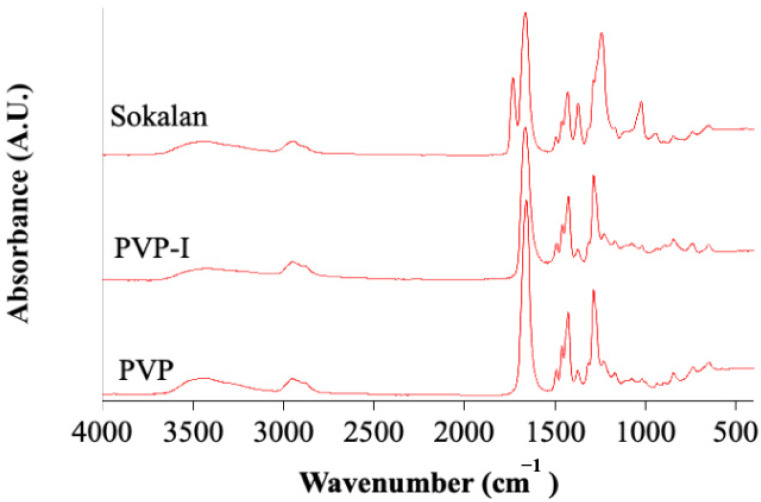
FTIR-ATR spectra of the minority components: PVP, PVP-I, and Sokalan.

The three minor components (PVP, PVP-I, and Sokalan; Figure 1), introduced into the PP matrix have different chemical-physical characteristics, as highlighted by the infrared spectra of the samples (Figure 3).

**Figure 4:**
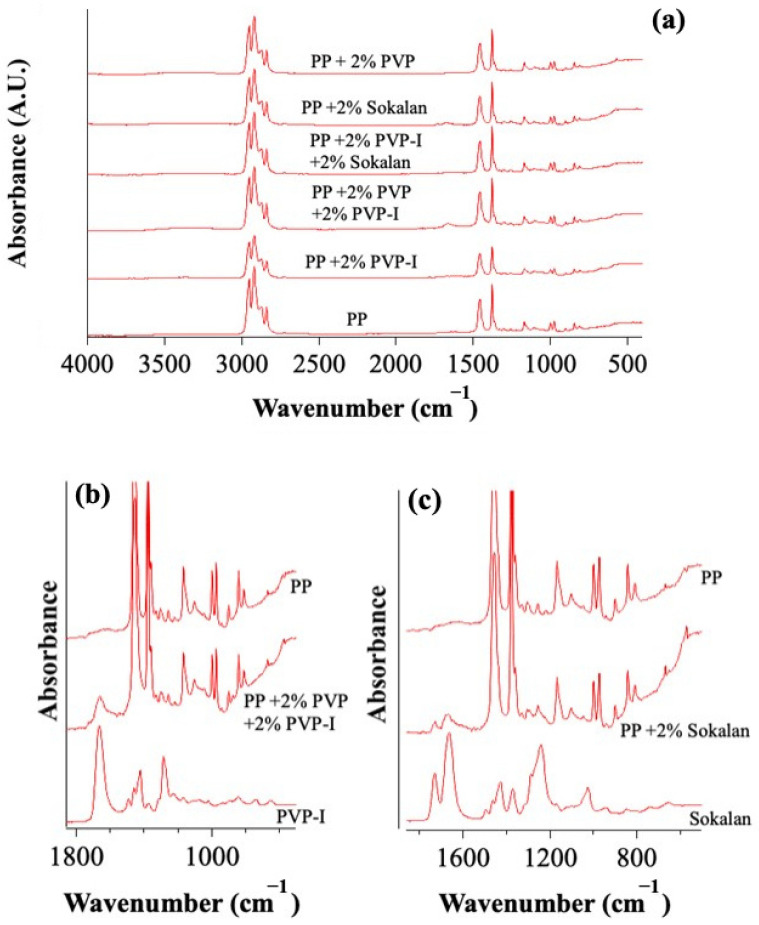
(**a**) FTIR-ATR spectra of PP and blends. (**b**,**c**) FTIR-ATR spectra of the fingerprint region of the PP + 2% PVP + 2% PVP-I, and PP + 2% Sokalan samples in comparison with that of the PP, PVP-I, and Sokalan components.

The PVP and PVP-I samples exhibited typical absorptions of poly(N-vinylpyrrolidone) at 3000–3500 cm^−1^ (OH str.), 2932, 2953, and 2954 cm^−1^ (C-H str.), 1645 cm^−1^ (C=O str.), 1288 cm^−1^ (C-N bend.), 1430 cm^−1^ (C-H bend.), and 1018 and 568 cm^−1^ (CH_2_ rock. and N-C=O bend.) [19,20]. Furthermore, in the PVP-I spectrum, a slightly stronger absorption can be observed in the tail of the C=O str. peak between 1600 and 1550 cm^−1^, possibly due to the str. of C=C groups. The presence of these groups may be due to oxidation processes, which can cause a loss of pyrrolidone rings from the polymer backbone and the formation of conjugated structures [21]. The Sokalan sample presented, in addition to PVP absorptions, intense peaks at 1725 (C=O str.), 1365 (CH_3_ bend.), 1232 (C-O str.), 1021 (C-O str.), and 933 cm^−1^ (C-H bend.), which are assignable to an ester [22], confirming the presence of a copolymer. The six blends showed all the characteristic absorptions of PP (Figure 4): 2950, 2915, and 2838 (CH str.), 1455 (CH_2_ bend.), 1377 (CH_3_ bend.), 1166 (CH bend., CH_3_ rock., and C-C str.), 997 (CH_3_ rock., CH_3_ bend., and CH bend.), 972 (CH_3_ rock. and C-C str.), 840 (CH_2_ rock. and C-CH_3_ str.), and 808 cm^−1^ (CH_2_ rock., C-C str., and C-CH str.) [22]. The spectrum of PP also showed weak and broad absorptions in the 1725–1700-cm^−1^ range, ascribable to oxidation products from limited thermal oxidation, which can typically occur in the processing of polyolefins. In addition, the PP + 2% PVP + 2% PVP-I and PP + 2% Sokalan samples also showed absorptions in the fingerprint region attributable to the additional components, as highlighted in Figure 4. In particular, the PP + 2% PVP + 2% PVP-I sample, exhibiting an amber color, had absorptions attributable to the PVP and PVP-I components. Unsurprisingly, the PP + 2% Sokalan sample has absorptions attributable to the Sokalan component. However, the spectrum of the PP + 2% PVP-I + 2% Sokalan samples did not show any additional absorptions compared with those of the PP. Spectral shifts passing from the pure PP to the blends were either not detected or extremely close to the spectral resolution of 4 cm^−1^. Considering that PP was expected to give mainly dispersion forces with the additives, some band broadening rather than peak shifts could have been expected. However, this was not observed, likely because the percentage of additives was too low.

**Figure 5:**
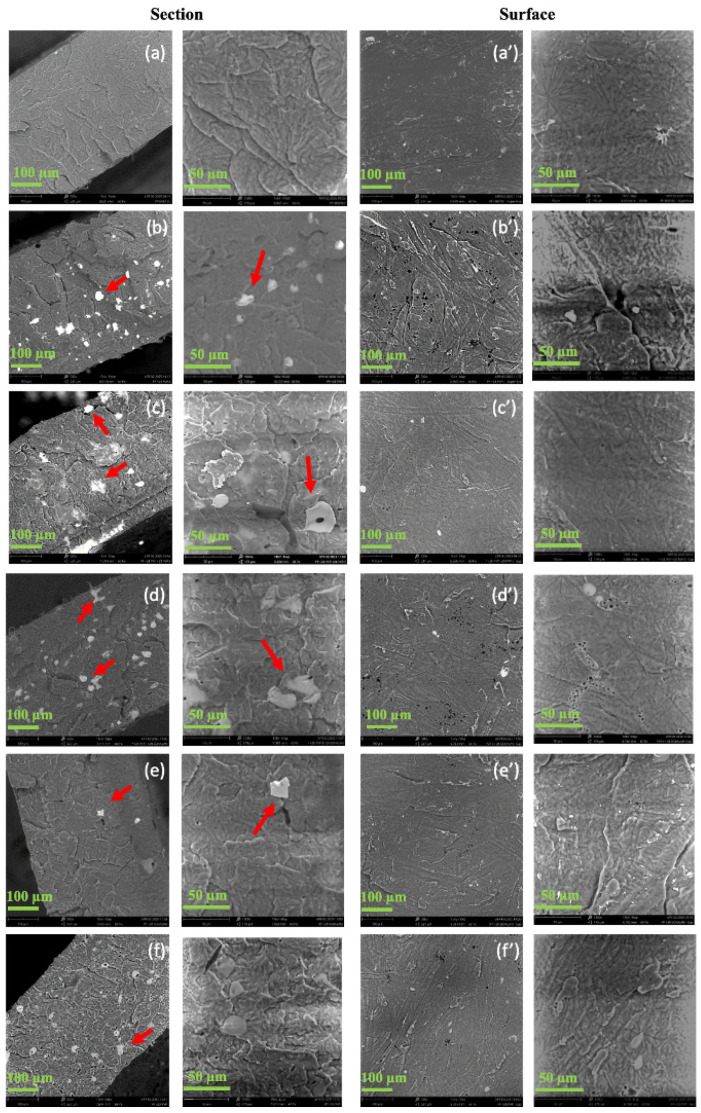
SEM images of fractured cross-section and surface of (**a**,**a’**) PP, (**b**,**b’**) PP + 2% PVP-I, (**c**,**c’**) PP + 2% PVP + 2% PVP-I, (**d**,**d’**) PP + 2% PVP-I + 2% Sokalan, (**e**,**e’**) PP + 2% Sokalan, and (**f**,**f’**) PP + 2% PVP samples.

The morphological analysis of the polymer blends (surface and section) was evaluated to assess the effect of the additives to the PP matrix (Figure 5). The surface analysis (Figure 5a’–f’) showed no significant differences due to the presence of additives in comparison with the PP sample. Instead, the fractured cross-section analysis (Figure 5a–f) showed important variability in the examined samples. In almost all the blends studied, the presence of aggregates was found in the bulk of the matrix. In particular, the blends with 2% PVP-I (both binary and ternary) were characterized by a greater segregation of the particles in the innermost part of the sample, even if, in both ternary blends, agglomerates were also present adjacent to the surface of the respective samples (Figure S1). The dimensions of the aggregates were variable, being between 20 and 50 microns. The small aggregates observed within the bulk of the PP polymer matrix appeared to be numerous, well dispersed, and spatially separated, as shown in the SEM micrographs at higher magnifications (Figure 5). The fracture surfaces at higher magnifications appeared homogeneous, showing a uniform structure without significant holes and limited discontinuities. Overall, SEM showed good adhesion between the different phases, which was similar for all the blends.

### 3.2. Thermal Properties

**Figure 6:**
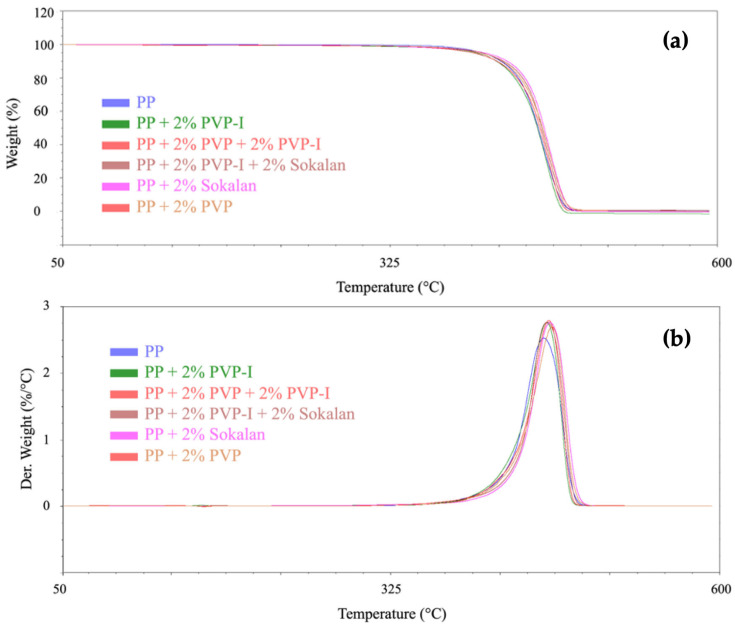
(**a**) TGA and (**b**) DTG curves of PP and its blends.

TGA and differential thermogravimetry (DTG) were used to investigate the influence of the minor components on the thermal stability and decomposition properties of the samples. The PVP sample showed weight loss of about 9% up to 250 °C, due to the release of water that was originally absorbed by this highly hydrophilic polymer. The peak in the DTG curve at 200 °C was ascribed to the dehydration of lower molecular weight polymer chains or impurities in the sample [23]. The most intense thermal event started over 250 °C and was centered at 450 °C (Figure S2a) due to the almost complete pyrolysis of the polymer, as reported in the literature [23,24]. The PVP-I sample had significantly different thermal behavior, with relevant events at 220 °C, 310 °C, and 400 °C, in addition to the loss of water within 100 °C (Figure S2b). The multiple pyrolysis peaks at lower temperatures than PVP were consistent with the presence of conjugated structures (likely formed from oxidized products) also suggested by ATR-FTIR, which are thermally sensitive. The Sokalan sample showed two clearly separate thermal events at 330 °C and 440 °C (Figure S2c), ascribable to the deacetylation of PVAc and thermal degradation of PVP, respectively [24]. An increase in the thermal stability of the blends compared with the PP sample was recorded via TGA (Figure 6). The samples showed limited weight loss below 250 °C, with values spanning from ca. 0.1% (PP) to 0.6% for the composite systems. At the end of the temperature ramp, almost all the samples were completely pyrolyzed, with residue values lower than two percentage points (Table 2). Interestingly, for all the blends, the maximum decomposition temperature was higher than that of PP (Figure 6b).

DSC thermograms were recorded to study the effect of the minor components on the main thermal properties and crystallinity (Table 3). The melting temperature (***Tm***) did not change significantly across the series of pure and blended systems, remaining at 170 ± 2 °C, according to the literature [25,26]. As expected, neither of the two PVP and PVP-I compounds displayed a DSC signal related to melting, as PVP and PVP derivatives are usually amorphous. The DSC curves acquired during the first and second heating ramps were significantly different for all the samples. In fact, the PP + 2% PVP-I and PP + 2% PVP + 2% PVP-I samples also showed a second, smaller melting peak at ca. 150 °C only in the first heating ramp. The ***Tm*** values were consistent with those obtained by DTG and in agreement with the literature [25,26]. The decrease in melting enthalpy and crystallinity following thermal history erasure could be explained by considering the fast cooling rate after the first heating ramp. Nevertheless, the crystallinity of the blends was always advantageously higher than that of PP. Numerous small, well-dispersed agglomerates in the polymer matrix (Figure 5) act as nucleation centers, leading to an increase in the crystallinity of the composites compared with the neat PP. Their relatively high number provided an increase in the density of heterogeneous nucleation sites, which is consistent with the higher crystallinity observed in the composites compared with the neat polymer [27]. The irregular morphology of the aggregates further enhanced this effect, since non-spherical or rough surfaces present a larger effective interfacial area that favors polymer–filler interactions. The combination of a small aggregate size, homogeneous spatial distribution, and irregular surface morphology appears to contribute synergistically to the nucleating effect, thereby explaining the enhanced crystallinity of the investigated samples.

### 3.3. Mechanical Properties

Blending polymers with different chemical structures is a usual method in academia and industry for producing materials with resultant properties better than those the single component shows. The main question with polymer blends is their miscibility, which is critical for chemically different polymers that create immiscible blends. This blending of incompatible polymers leads to materials with weak bonding strength and low mechanical integrity, which can be improved by introducing a third component able to increase the compatibility between the constituents of polymeric blends. This approach generally determines the modification of the adhesion properties at the interfacial boundary of the immiscible components through interphase formation [28,29]. The effect on the mechanical characteristics and morphology due to the introduction of PVP to binary polymeric blends, based on PP and polyethylene, was studied by Salihet al. [30]. They found enhanced mechanical properties for the prepared composites when the PVP material was blended within limited ratios (4–8%). Accordingly, a low percentage of minor components was added to not alter the mechanical properties of the PP polymeric material. All the mechanical properties, namely the elastic modulus (***E***), tensile strength (***TS***), and elongation at break (***EB***), investigated for PP and its blends, are reported in Table 4.

In general, as was our aim, the changes induced by the blending were limited. The presence of the additives slightly stiffened the blend samples, possibly due to the higher crystallinity, as evidenced by a small increase in the elastic modulus, making them less flexible. The tensile strength at break decreased moderately, mainly because of the decrease in the elongation at break, ranging from 7.2 to 6.0% for the blends, with lower values for the ternary ones (Table 4). The elongation at break can be considered a good indicator of the compatibility of the system. In fact, it is highly sensitive to both structural and morphological variations in the polymer system. Therefore, when examining the data reported in Table 4, the PP + 2% PVP-I, and PP + 2% PVP samples, having the same nominal percentage of dispersed particles, seemed to have the best adhesion. This result could appear to disagree with the SEM analysis, which did not highlight differences among the blends. However, SEM images are limited to microscopic areas, while tensile testing is an averaging analysis involving an extensive portion of the samples. The introduction of both compatibilizers (ternary blend samples PP + 2% PVP + 2% PVP-I and PP + 2% PVP-I + 2% Sokalan) provides a reduction in the elongation at break and less adhesion [31].

### 3.4. Wettability of the Film Surfaces and the SCA

**Figure 7:**
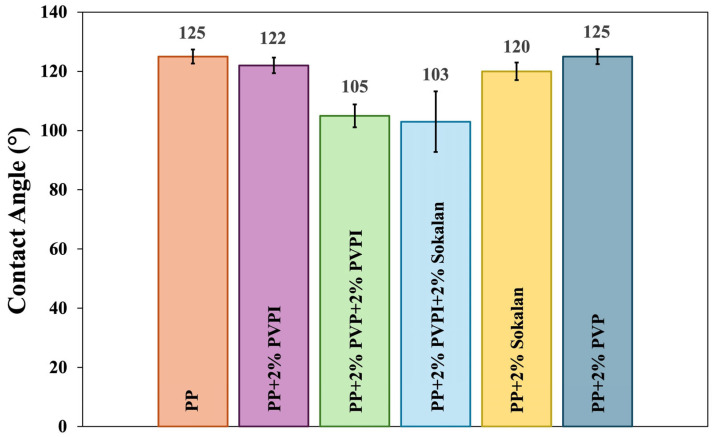
SCA of PP and its blends.

PVP, PVP-I, and Sokalan are all hydrophilic polymers, unlike PP, which is hydrophobic. To check the effects of blending with minor hydrophilic components on the PP surface wettability, a comparative static contact angle (SCA) analysis was carried out. As shown in Figure 7, the SCA of the ternary blend samples (PP + 2% PVP + 2% PVP-I and PP 2% PVP-I + 2% Sokalan) decreased compared with neat PP. While for the other samples (PP + 2% PVP-I, PP + 2% Sokalan and PP + 2% PVP) the wettability value remained almost unchanged in relation to neat PP because of the low percentage of additives mainly dispersed in the bulk, instead, greater variability in the data, reflected by the higher standard deviation, was obtained for the samples with a higher percentage of hydrophilic components, which could be due to a marked surface roughness or, more reasonably, aggregation of hydrophilic components, which were present in higher amounts and in the sheet surface [32]. The wettability analysis agrees with the SEM morphological analysis of the samples (Figure 5). The presence of a higher concentration of particle aggregates inside the matrix bulk led to concordance with the values recorded in the case of the PP sample. Thus, only for the ternary blends, the presence of agglomerates of the hydrophilic components, which were also found on the surface (Figure 5c,d), could be responsible for the lower SCA values for these two samples.

### 3.5. Barrier Properties

**Figure 8:**
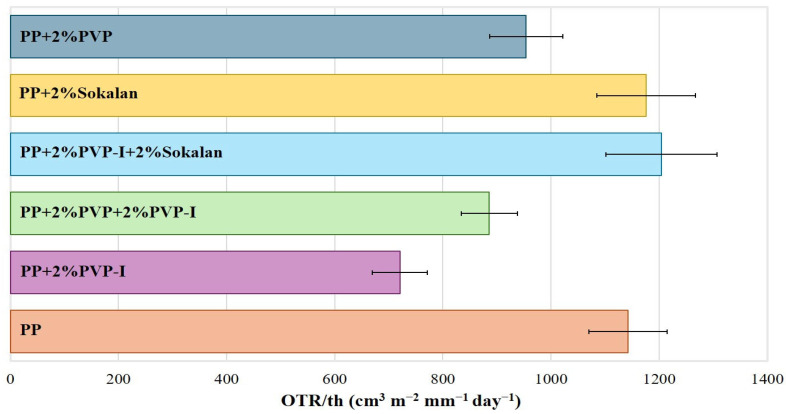
Average OTR/th values for the PP and its blends.

Table S1 shows the experimental values of the oxygen transmission rate (OTR) of the sheets obtained via compression molding. All of the blends showed OTR values lower than those measured for the pure PP matrix. However, because of the slightly different values for the thickness of the specimens, the OTR values were normalized with respect to that of the sample sheet (OTR/th, Table S1). Figure 8 shows the trend of the OTR/th for all samples. The PP + 2% PVP-I blend, with a higher density of agglomerates (Figure S1) and enhanced degree of crystallinity (60%, Table 3), displayed the lower OTR/th and a better oxygen barrier property than those of the PP matrix. On the contrary, both the binary and ternary blends containing the Sokalan showed a higher OTR/th than PP and the worst O_2_ barrier properties. As is well known, the diffusion of oxygen into the crystallites of semicrystalline polymers is much slower. In fact, the crystallites increase the tortuosity of the transport path, and a higher crystallinity (Table 3) can influence the oxygen barrier properties [33,34]. However, the presence of agglomerates (Figure 5) also affects the oxygen barrier properties because of their compact structure, being less permeable to gas diffusion. In addition, they contribute to creating longer and more intricate paths for oxygen molecules traveling through the sample [35,36]. The values found via OTR analysis show that the blend with the highest density of agglomerates (Figure S1) had a stronger barrier effect than the PP sample, which showed the lowest degree of crystallinity (Table 3). Only in the case of the samples with added Sokalan (both binary and ternary) was the barrier effect comparable or worse than that of the PP sample, probably due to the lower density of agglomerates and reduced adhesion between the components also found through the mechanical properties.

**Figure 9:**
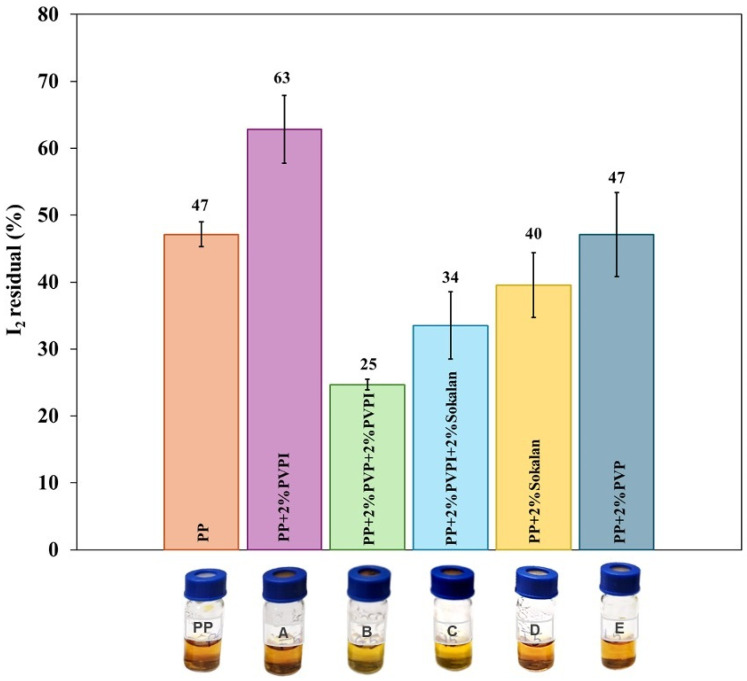
Residual molecular iodine (%) determined in PVP-I buffered solution after 5 days at 40 °C in vials closed with cups of PP and its blends.

**Figure 10:**
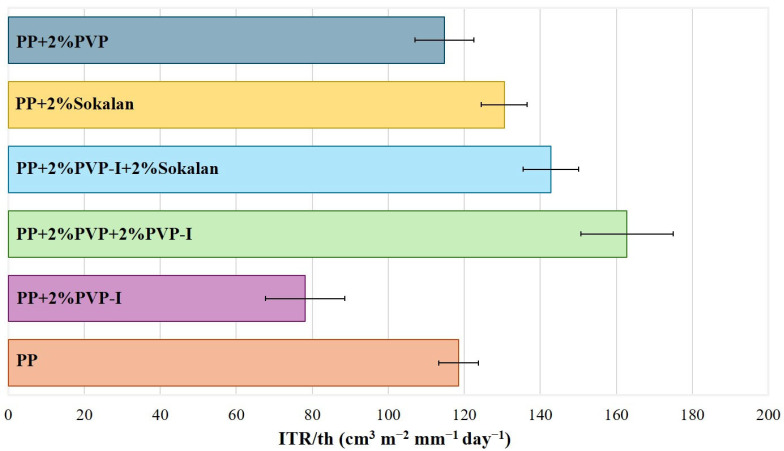
Average ITR/th values of PP and its blends.

To assess the molecular iodine barrier effect, comparative tests were carried out using gas chromatography vials in which the septum was replaced by laboratory-made discs in neat PP and in the respective blends (Figure 2). Figure 9 shows the percentage variation of the I_2_ concentration in the PVP-I solutions contained in the sealed vials with PP discs and their blends, whereas Figure 10 reports the data converted into the I_2_ transmission rate (ITR), normalized with respect to the thickness of the blend sheets. The amount of I_2_ in the solutions decreased for all samples. However, the residual I_2_ percentage in the PVP-I solution sealed by the neat PP discs was lower than that found in the PP + 2% PVP-I solution. This system therefore provided an I_2_ barrier effect, reducing its leaching compared with the neat PP (Table S2, Figure 10). PP + 2% Sokalan and PP + 2% PVP absorbed I_2_ vapor from the buffered PVP-I solution, evidenced by the color change. However, they did not retain it and did not provide a valuable barrier effect compared with the unmodified PP sample, as observed for similar PP-based systems with CX additives [15]. The permeability of gas molecules is usually expected to be a combination of two main processes, i.e., solubility and diffusion. Reasonably, in the blends containing PVP or Sokalan, which were able to complex the I_2_, their presence induced higher solubility in the polymeric discs, but the complex in a solid phase was formed in a limited amount. Thus, chemical interactions were reduced, and the iodine diffusion and release outside were enhanced.

**Figure 11:**
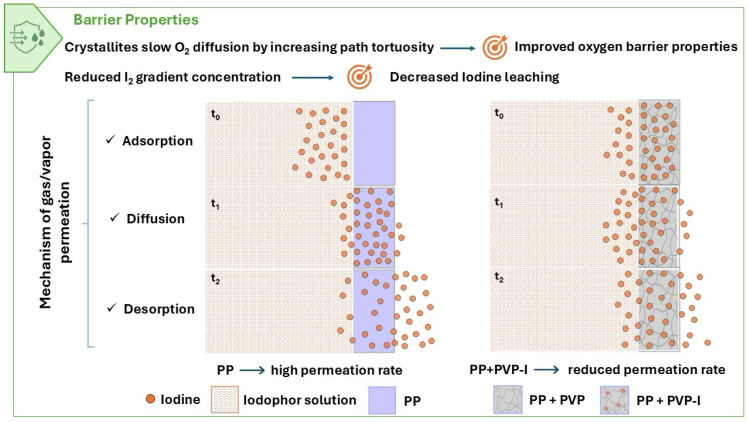
Schematic representation of the I_2_ leaching through PP, PP + PVP and PP + PVP-I barrier materials.

In particular, the typical mechanism for gas and vapor permeation through a barrier involves three steps: (1) absorption of the gas or vapor permeant molecules from the feed stream into the surface, (2) dissolution and then diffusion through the barrier material, and (3) desorption on the permeate side of the barrier from the surface into the low-concentration vapor phase. This solution-diffusion mechanism is driven by a concentration gradient. It depends on the chemical potential difference of the permeant across the barrier. The temperature, the chemical nature of the membrane material, and the permeant, as well as the molecular interactions between the permeating species and the membrane material, are key factors influencing permeation as well [37]. In Figure 11, we show a schematic representation of the mechanism of I_2_ permeation through the PP material and the PP blends. According to our approach, the introduction of a small amount of PVP-I reduced the iodine gradient concentration, decreasing the I_2_ leaching in the absorption and diffusion steps. Furthermore, the inclusion of PVP-I provided interactions with the iodine diffused in the polymeric barrier, slowing down the I_2_ desorption rate. Furthermore, similar to what was described above for the oxygen barrier, the presence of PVP-I increased the tortuosity of the diffusion path, hindering gas permeation. This is due to a reduced diffusion gradient and specific interactions between the PVP-I and the iodine molecules. These results confirm that the barrier’s efficiency is determined by a synergistic interaction between the crystallinity, agglomerate morphology, concentration gradient and specific chemical interactions with permeants.

To monitor variation in the discs used in the I_2_ leaching test, UV-Vis reflectance spectra were recorded using a Jasco 750V UV-Vis spectrophotometer and a powder sample holder at time zero and after 5 days at 40 °C. The reflectance spectra of PP and its blends’ discs samples at t = zero and after 5 days at 40 °C are reported in Figure S3. The PP spectrum agrees with the literature [15,38]. The spectra of PP, PP + 2% Sokalan, and PP + 2% PVP after 5 days at 40 °C show a significant decrease in the reflectance percentage in the molecular iodine absorption region (~380 nm). As previously observed, this is indicative of the absorption of I_2_ vapor from the PVP-I buffered solution, which is also supported by the yellow dyeing of the disc samples [15]. However, in such cases, this did not provide less I_2_ leaching.

## 4. Conclusions

Restraining I_2_ leaching from plastic containers is quite a challenge. To test new approaches to reducing the loss of molecular iodine from an iodophor solution, we introduced small amounts of I_2_ to the polymeric material commonly used in eye bottle droppers, i.e., PP. Thus, PP was blended with PVP-I or with a second polymeric component (PVP or Sokalan) able to complex the I_2_ released from an iodophor solution. The PP and blends sheets were characterized for surface and bulk changes by contact angle, SEM, TGA, DSC, ATR-FTIR, tensile test, oxygen permeability and UV-Vis diffuse reflectance spectra. SEM analysis showed good adhesion between the different phases, being comparable for all the blends. A comparative study showed that the minority components in the blends increased thermal stability as well as crystallinity. Moreover, the PP blends with PVP-I prompted higher oxygen barrier properties and a reduction in I_2_ leaching in comparison with PP. Overall, the preliminary results highlight that PVP-I polyolefin blends could be a promising novel approach to enhancing the shelf life of iodine-based formulations. Further studies are underway comparing other blends with iodophor components.

## Figures and Tables

**Table 1:** PP-based blends with and without PVP, PVP-I, and Sokalan.

Abbreviation	Composition
PP	Polypropylene
PP + 2% PVP-I	98% polypropylene + 2% povidone-iodine
PP + 2% PVP-I + 2% PVP	96% polypropylene + 2% povidone-iodine + 2% poly(N-vinylpyrrolidone)
PP + 2% PVP-I + 2% Sokalan	96% polypropylene + 2% povidone-iodine + 2% vinyl pyrrolidone vinyl acetate 55/45
PP + 2% Sokalan	98% polypropylene + 2% vinyl pyrrolidone vinyl acetate 55/45
PP + 2% PVP	98% polypropylene + 2% poly(N-vinylpyrrolidone)

**Table 2:** DTG and DSC data for PP and its blendsxc.

Sample	WL_50°C−250°C_ (%)	WL_250°C−550°C_ (%)	*Tp* (°C) *	*Tm* (°C) *
PP	0.1	99.5	453	170
PP + 2% PVP-I	0.6	100.0	455	168
PP + 2% PVP + 2% PVP-I	0.6	99.0	457	167
PP + 2% PVP-I + 2% Sokalan	0.4	99.0	456	168
PP + 2% Sokalan	0.4	100.0	458	168
PP + 2% PVP	0.5	99.0	460	169

* Experimental error within the instrument accuracy, i.e., 1 °C.

**Table 3:** DSC data for PP and its blends.

First Heating Ramp			
**Sample**	*Tm* (°C) *	***∆Hm* (J/g)**	***Xc* (%)**
PP	168	116	56
PP + 2% PVP-I	166	124	60
PP + 2% PVP + 2% PVP-I	165	123	59
PP + 2% PVP-I + 2% Sokalan	168	122	59
PP + 2% Sokalan	166	127	61
PP + 2% PVP	167	132	64
**Second Heating Ramp**			
**Sample**	*Tm* (°C) *	***∆Hm* (J/g)**	***Xc* (%)**
PP	167	114	55
PP + 2% PVP-I	168	119	58
PP + 2% PVP + 2% PVP-I	167	120	58
PP + 2% PVP-I + 2% Sokalan	167	120	58
PP + 2% Sokalan	166	119	58
PP + 2% PVP	166	120	58

* Experimental error within the instrument accuracy, i.e., 1 °C.

**Table 4:** Mechanical properties of the PP-based samples.

Sample	*E* [MPa]	*TS* [MPa]	*EB* [%]
PP	950.0 ± 16.0	28.6 ± 1.3	7.8 ± 0.8
PP + 2% PVP-I	980.3 ± 46.6	25.8 ± 0.8	7.2 ± 0.6
PP + 2% PVP + 2% PVP-I	1008.7 ± 24.5	24.8 ± 1.1	6.0 ± 0.6
PP + 2% PVP-I + 2% Sokalan	1050.4 ± 33.7	25.7 ± 1.7	6.3 ± 0.6
PP + 2% Sokalan	1042.4 ± 69.3	26.0 ± 1.8	6.8 ± 0.6
PP + 2% PVP	988.6 ± 28.5	25.4 ± 2.5	7.2 ± 0.6

## Data Availability

The data presented in this study are available on request from the corresponding authors.

## Supplementary Materials

The following supporting information can be downloaded at: https://www.mdpi.com/article/10.3390/polym17182442/s1, Figure S1: Spatial distribution of aggregates in the bulk of the samples, obtained from the SEM images of the fractured sections by counting the aggregates and normalized per unit of surface area analyzed; Figure S2: TGA/DTG curves of (a) PVP, (b) PVP-I and (c) Sokalan; Figure S3: Reflectance (%) spectra and photographic documentation of discs of PP and its blends at t = 0 (blue line) and after 5 days (orange line) at 40 °C; Table S1: Oxygen transmission rate (OTR) of PP-based samples; Table S2: Iodine transmission rate (ITR) of PP-based samples.
