# Supplementary material for: PP-Based Blends with PVP-I Additive: Mechanical, Thermal, and Barrier Properties for Packaging of Iodophor Pharmaceutical Formulations
Source: Polymers (Basel). 2025 Sep 9;17(18):2442. doi: 10.3390/polym17182442 (PMC12473595; doi:10.3390/polym17182442)
Supplement: Supplementary file 1 [file polymers-17-02442-s001.zip › polymers-3809659-supplementary.pdf]

Supplementary Materials

PP based blends with PVP-I additive: mechanical, thermal and barrier properties for packaging of iodophor pharmaceutical formulations

Melania Leanza [1], Domenico Carmelo Carbone [1], Giovanna Poggi [2], Marco Rapisarda [1], Marilena Baiamonte [3,4], Emanuela Teresa Agata Spina [1], David Chelazzi [2], Piero Baglioni [2], Francesco Paolo La Mantia [3,4,*] and Paola Rizzarelli [1,*]

1 Institute of Polymers, Composites and Biomaterials - National Research Council (IPCB-CNR), 95126 Catania, Italy; melanialeanza@cnr.it (M.L.); domenicocarmelo.carbone@cnr.it (D.C.C.); marco.rapisarda@cnr.it (M.R.); emanuelateresaagata.spina@cnr.it (E.T.A.S.)
2 Department of Chemistry "Ugo Schiff" and CSGI, University of Florence,
50019 Sesto Fiorentino, Florence, Italy; poggi@csgi.unifi.it (G.P.); david.chelazzi@unifi.it (D.C.); piero.baglioni@unifi.it (P.B.)
3 Department of Engineering, University of Palermo, 90128 Palermo, Italy; marilena.baiamonte@unipa.it (M.B.)
4 National Interuniversity Consortium of Materials Science and Technology (INSTM),
50121 Florence, Italy
* Correspondence: francescopaolo.lamantia@unipa.it (F.P.L.M.); paola.rizzarelli@cnr.it (P.R.)

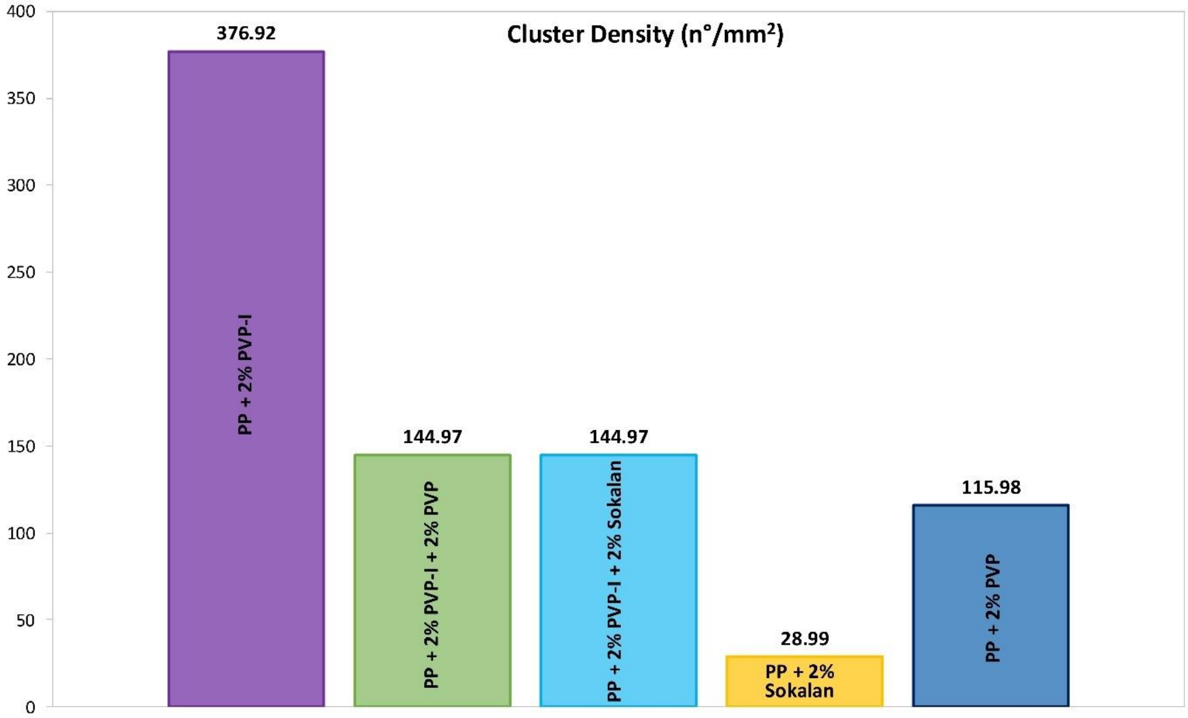

Figure S1. Spatial distribution of aggregates in the bulk of the samples, obtained from the SEM images of the fractured sections by counting the aggregates and normalized per unit of surface area analyzed.

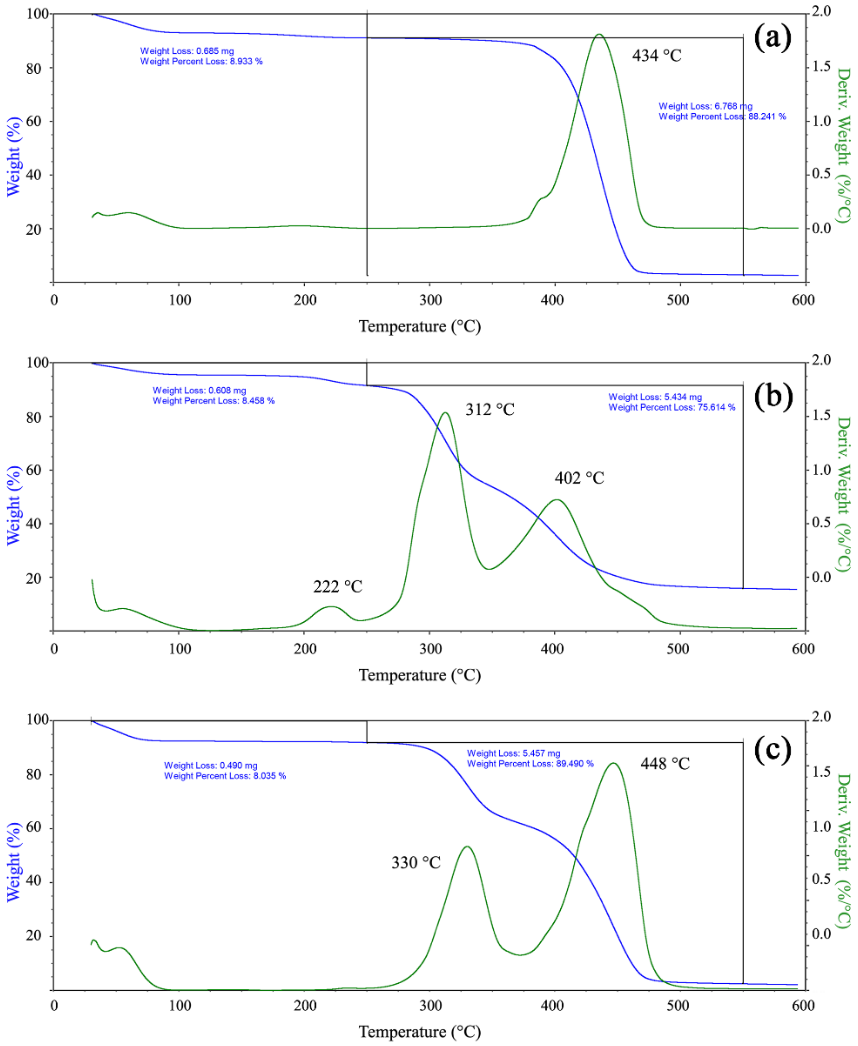

Figure S2. TGA/DTG curves of **(a)** PVP, **(b)** PVP-I and **(c)** Sokalan.

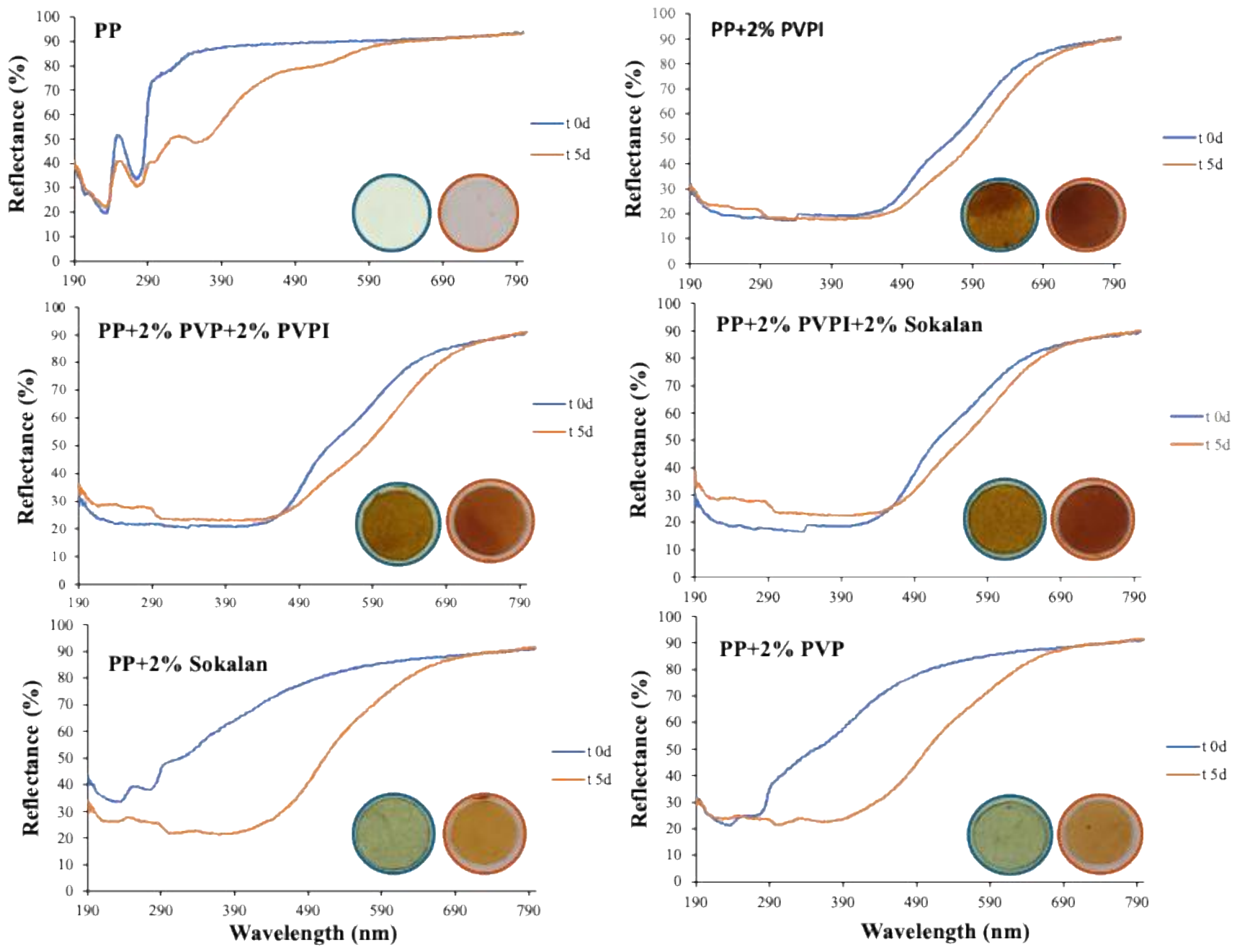

Figure S3. Reflectance (%) spectra and photographic documentation of discs of PP and its blends at t = 0 (blue line) and after 5 days (orange line) at 40 °C.

Table S1. Oxygen transmission rate (OTR) of PP-based samples.

Sample	OTR (cm^3 m^{-2} day^{-1})	OTR/th (cm^3 m^{-2} mm^{-1} day^{-1})
PP	297 ± 18	1142 ± 72
PP + 2% PVP-I	209 ± 15	721 ± 51
PP + 2% PVP + 2% PVP-I	217 ± 13	886 ± 52
PP + 2% PVP-I + 2% Sokalan	283 ± 23	1204 ± 103
PP + 2% Sokalan	294 ± 21	1176 ± 91
PP + 2% PVP	229 ± 16	954 ± 68

Table S2. Iodine transmission rate (ITR) of PP-based samples.

Sample	ITR (cm^3 m^{-2} day^{-1})	ITR/th (cm^3 m^{-2} mm^{-1} day^{-1})
PP	54 ± 2	119 ± 5
PP + 2% PVP-I	38 ± 5	78 ± 10
PP + 2% PVP + 2% PVP-I	79 ± 1	163 ± 12
PP + 2% PVP-I + 2% Sokalan	67 ± 3	143 ± 7
PP + 2% Sokalan	62 ± 1	130 ± 6
PP + 2% PVP	54 ± 4	115 ± 8
